# Examining the relationship between meeting 24-hour movement behaviour guidelines and mental health in Chinese preschool children

**DOI:** 10.3389/fped.2024.1337158

**Published:** 2024-03-18

**Authors:** Long Yin, Fang Li, Pan Liu, Zhiqiang Yin, Zongyu Yang, Linchun Pi, Zan Gao

**Affiliations:** ^1^ College of Physical Education, Hunan Normal University, Changsha, China; ^2^ School of Physical Education, Hunan First Normal University, Changsha, China; ^3^ School of Physical Education, Hunan University of Technology, Zhuzhou, China; ^4^ English Course Group, Hengyang First High School, Hengyang, China; ^5^ School of Physical Education and Health Sciences, Guangxi University for Nationalities, Nanning, China; ^6^ School of Sports Science, Hengyang Normal University, Hengyang, China; ^7^ Department of Kinesiology, Recreation, and Sport Studies, The University of Tennessee, Knoxville, TN, United States

**Keywords:** 24-h Movement Behaviour, externalizing problems, internalizing problems, light-intensity physical activity, moderate-to-vigorous physical activity, prosocial behaviour, sedentary behaviour, screen time

## Abstract

**Background:**

Limited research has explored the relationship between adhering to 24-h Movement Behaviour guidelines and mental health in Chinese preschool children. The objectives of this study encompassed two primary goals: (1) to investigate the adherence of preschool children in China to the 24-h Movement Behaviour guidelines; and (2) to analyze the relationship between fulfilling various combinations of these guidelines and mental health, identifying the most advantageous combination.

**Methods:**

Utilizing a convenience sampling approach, this study included 205 preschool children (117 boys and 88 girls, average age 4.8 ± 0.51 years) from five kindergartens in Hengyang, Hunan Province. The physical activity (PA) and sedentary behaviour of preschool children were objectively assessed using waist-worn accelerometers, while sleep duration and screen time were reported by the children's parents. To evaluate mental health, the parent version of the internationally validated Strength and Difficulties Questionnaire (SDQ) was employed, which measures externalizing problems, internalizing problems, and prosocial behaviour. Employing Mplus 8.0 for Structural Equation Modeling analysis, while controlling for demographic variables, the study explored the connection between preschool children's mental health and their adherence to the 24-h Movement Behaviour guidelines.

**Results:**

Worryingly, merely 14.6% of preschoolers met the recommended guidelines for all three aspects (PA, sleep duration, and screen time). Positive correlations were identified between meeting PA guidelines and displaying prosocial behaviour (*β* = 0.184; *p* < 0.05), while screen time adherence exhibited a negative correlation with externalizing problems (*β* = −0.207; *p* < 0.05). Similarly, there was a negative association between sleep duration adherence and externalizing problems (*β* = −0.191; *p* < 0.05). Meeting all three recommended guidelines was notably linked to enhanced prosocial behaviour (*β* = 0.464; *p* < 0.05), while following the screen time and sleep duration guidelines was negatively associated with externalizing problems (*β* = −0.246; *p* < 0.05).

**Conclusion:**

This study underscores the limited adherence of Chinese preschoolers to the comprehensive 24-h Movement Behaviour guidelines. Noteworthy findings include the positive influence of PA on prosocial behaviour, alongside the significant roles that sleep duration and screen time play in mitigating externalizing problems within this age group. Alignment with the 24-h Movement Behaviour guidelines is associated with more favorable mental health indicators in preschoolers.

## 1 Introduction

Mental health encompasses a condition of both physical and psychological well-being, facilitating efficient functioning that effectively manages external stressors and makes positive contributions to society (1). However, research indicates that an alarming 8%–10% of children under the age of 5 face mental health challenges, manifested in various forms. These challenges range from internalizing behaviors like anxiety and depression, to externalizing behaviors such as aggression, as well as neurodevelopmental disorders, notably attention-deficit/hyperactivity disorder (2). These concerns can arise early in life, potentially influencing the child's future state of well-being (3).

Appreciating the significance of mental well-being, the Chinese Ministry of Education has taken proactive measures through the implementation of the “Special Action Plan for Comprehensively Enhancing and Enhancing Students’ Mental Health in the New Era (2023–2025)” (4), which harmonizes with the “Integrated Mental Health Action Plan 2013–2030” (5). This endeavor underscores the nationwide acknowledgment of mental health as a primary concern, thereby triggering heightened focus on the mental welfare of Chinese preschoolers. The emphasis lies in pinpointing factors that exert influence and formulating efficacious interventions. There exists a multitude of factors that exert an influence on mental well-being, and among these, moderate-to-vigorous physical activity (MVPA) stands out for its established benefits on the mental health, motor skills, and cognitive development of preschool children (6, 7). Furthermore, some research indicates that even light-intensity physical activity contributes positively to children's mental health (8). Beyond this, sufficient sleep duration has demonstrated the capacity to diminish the prevalence of anxiety and depression among children and adolescents (9, 10). Conversely, screen-based sedentary behaviour, encompassing activities such as television, tablet, and computer usage, has the potential to exert adverse effects on both physical and mental well-being (11, 12).

However, past research has primarily examined individual components such as physical activity (PA), sedentary behaviour, and sleep duration, scrutinizing their connections with specific facets of mental health, often overlooking the intricate interplays between these three behaviours (13, 14). Certain academics recognize the significance of holistically considering the cumulative impact of multiple behaviours, encompassing PA, sleep duration, and sedentary behaviour (15–17). Recent investigations have underscored that young children engage in sleep duration, sedentary behaviour, and PA throughout the entirety of the 24-h day, collectively termed as 24-h Movement Behaviour (18).

According to the guidelines provided by the World Health Organization, children aged 3–6 should engage in 3 h daily PA, including 60 min of MVPA. For children aged 3–4, daily screen time should be limited to 1 h, with a recommended sleep duration of 10 to 13 h per day. For children aged 5–6, daily screen time should be limited to 2 h, along with 9–11 h of sleep duration per day (19). Studies indicate that youngsters and teenagers who conform to these 24-h Movement Behaviour guidelines exhibit enhancements in emotional and psychosocial well-being (20), elevated cognitive performance (21), and a diminished occurrence of anxiety disorders and depression (22).

Furthermore, indications propose that apart from the favorable outcomes associated with adhering to the prescribed 24-h Movement Behaviour guidelines concerning the physical and mental health of young children, diverse amalgamations of strategies might produce differing impacts on well-being (23). Several studies have demonstrated that adherence to various configurations of the 24-h Movement Behaviour guidelines correlates with a reduced prevalence of depression and anxiety (22, 24, 25). Nonetheless, the literature does not uniformly ascertain which specific combinations of behaviors exhibit a stronger association with superior psychological functioning (22, 24, 25).Consequently, the identification of the most efficacious amalgamation of strategies for interventions aimed at mental health is of paramount importance (24).

Carson et al. identified specific combinations of PA + screen time and screen time + sleep duration linked to internalizing and externalizing problems among Canadian 3-year-olds (26). In a similar vein, Saunders et al. found that children and adolescents adhering to a low sedentary behaviour/high PA/sufficient sleep duration movement behaviour pattern displayed more favorable markers of obesity and cardiometabolism than those following a high sedentary behaviour/low PA/insufficient sleep duration pattern (23). However, it is worth noting that only a limited number of Chinese children and adolescents adhered to the recommendations outlined in the 24-h Movement Behaviour guidelines (7, 27). Concurrently, research has indicated that among 11 countries spanning five major geographical regions of the world, Chinese children exhibit the least average daily participation in MVPA (28). Despite potential variations in cultural norms and policies across different geographical locations, a comparative analysis with Japanese children, who are also from the Asian region, reveals that Japanese children have higher levels of physical activity than their Chinese counterparts (29). Furthermore, the prevalence of various mental disorders among Chinese children and adolescents surpasses that of other countries (30). Consequently, investigating the potential link between physical activity behaviors and mental health within the Chinese population becomes particularly pressing. To our understanding, only a handful of studies have integrated the effects of adhering to the 24-h Movement Behaviour guidelines on mental health in samples of Chinese preschool children.

Thus, the primary aim of this study was to investigate the correlation between adherence to the 24-h Movement Behaviour guidelines and the mental well-being of Chinese preschool children. To be more specific, our primary goals are as follows: (1) to scrutinize the degree of conformity with the 24-h Movement Behaviour guidelines among preschoolers in China, and (2) to delve into the connection between adhering to diverse combinations of the 24-h Movement Behaviour guidelines and mental health outcomes, pinpointing which amalgamation holds greater potential for yielding substantial advantages. Gaining insights into the influence of these Behaviours on mental health will play a pivotal role in shaping the formulation of effective interventions, aimed at bolstering the overall wellness of Chinese preschool children.

## 2 Methods

### 2.1 Design and participants

**Figure 1:**
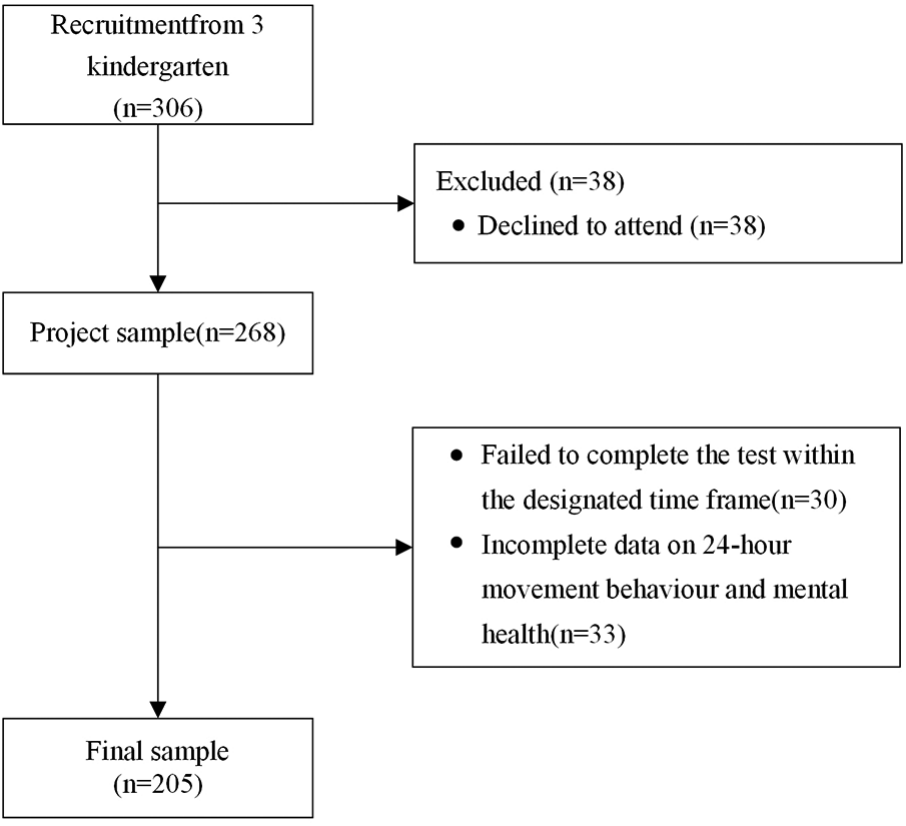
Process of recruitment and participant selection.

For this study, a total of 306 preschool children aged 3–6 years were recruited from five kindergartens situated in Hengyang City, Hunan Province, China, utilizing a convenience sampling approach. All these preschoolers were attendees of Early Childhood Education and Care Services (ECECs) sanctioned by the Hengyang Education Bureau, and they were without any chronic health conditions. Before taking part in the study, the parents of these children were provided with information about the research, and their formal consent was acquired through the completion of a signed informed consent form. However, consent was not provided by 38 parents, and an additional 30 children were unable to participate in the testing within the specified time frame. This process resulted in gathering valid responses from 238 participants. However, due to incompleteness, 33 of these responses were later deemed unsuitable for analysis and thus excluded. The final sample comprised 205 preschool-aged children, with an average age of 4.8 years (SD = 0.51 years), including 117 boys and 88 girls (see Figure 1).

### 2.2 Measures

In the 2019 WHO guidelines and the 2020 physical activity and sedentary behavior guidelines, a structured daily regimen for children aged 3–6 is advocated (19, 31). For children aged 3–4 years, it is recommended to engage in at least 180 min of PA daily, including 60 min of moderate to vigorous physical activity (MVPA), limit screen time to no more than 1 h, and ensure a sleep duration of 10–13 h per day. For children aged 5–6 years, the guidelines suggest at least 60 min of MVPA daily, restricting screen time to no more than 2 h, and a sleep duration of 9–11 h per day. These guidelines provide a valuable framework for our research.

#### 2.2.1 PA and sedentary behaviour

To obtain objective measurements of PA and sedentary behaviour among preschool children, a waist-worn accelerometer (Actigraph, model wGT3X-BT, USA), a validated instrument (32), was utilized. Kindergarten teachers and parents were provided instructions on the correct use of the accelerometer and were tasked with supervising the children while they wore it on a daily basis. The accelerometer was worn around the child's waist and right hip continuously for seven consecutive days, excluding times when the child was bathing, swimming, or sleeping. It recorded sedentary behaviour, light-intensity physical activity, moderate physical activity (MPA), and vigorous physical activity (VPA) in 15-second epochs specifically for preschoolers. Following the completion of the testing period, the accelerometers were initialized and the data were analyzed using ActiLife software (ActiGraph Corps., Pensacola, FL, USA; Version 6.13.3).

In this study, the PA intensity cut points proposed by Butte (2014) were applied, categorizing movement behaviour into sedentary behaviour at 239 counts/epoch, light-intensity physical activity in the range of 240–2,119 counts/epoch, MPA spanning 2,120–4,449 counts/epoch, and VPA at 4,450 counts/epoch (33). The total PA was computed by summing up light-intensity physical activity, MPA, and VPA, while MVPA was calculated by summing up MPA and VPA. To ensure valid accelerometer data, participants were required to wear the device for a minimum of 8 h per day on at least 3 valid days (consisting of two weekdays and one weekend day), in accordance with Choi et al.'s wear-time algorithm (34).

When considering sedentary behaviour in children, the primary focus is on screen time (35), which includes time spent using computers, TVs, and smartphones. To collect data on screen time, a questionnaire was provided to parents. The questionnaire contained the following question: “How much time does your child spend watching TV, using a computer, using a smartphone, or playing video games on weekday/weekends?” The average daily screen time was calculated using the formula: [(weekday screen time × 5 days] + [weekend screen time × 2 days)]/7 days.

#### 2.2.2 Sleep duration

Data on sleep duration were collected using a validated questionnaire completed by the parents of the selected children (36). Data was collected independently for weekdays and weekends, encompassing both bedtime and wake-up time. The total sleep duration was calculated using the following formula: [(weekday night sleep duration × 5 days + weekend night sleep duration × 2 days)/7 days + (weekday daytime sleep duration × 5 days + weekend daytime sleep duration × 2 days)/7 days]. If any irregularities were observed in the collected data, the researchers promptly communicated with the parents to confirm the information.

#### 2.2.3 Mental health

To evaluate the mental health of preschool children, this study employed the parent version of the Strength and Difficulties Questionnaire (SDQ), a widely recognized and validated instrument for assessing emotional issues, behavioral challenges, hyperactivity/inattention, peer relationship problems, and prosocial behavior in children aged 3–16 years (37, 38). The SDQ comprises 25 items divided across five subscales, each with five items. However, focusing on the study's specific aims, a three-factor model of the SDQ was adopted, concentrating on internalizing problems, externalizing problems, and prosocial behavior (39, 40). This model was specifically chosen for its applicability to a normal child population, where psychological health exhibits a normal variation range. It allows for an examination of how different scores within this normal range may still correlate with 24-h activity behaviors. The internalizing problems scale merges the emotional problems and peer relationship problems, while the externalizing problems scale encompasses the behavioral problems and hyperactivity/inattention subscales. Elevated scores in the internalizing and externalizing scales indicate increased psychological challenges, whereas higher scores in the prosocial behavior scale suggest enhanced psychosocial functioning. This adaptation was guided by prior literature endorsing the three-factor model's efficacy in general and low-risk child populations (39, 40), highlighting its relevance for our study's focus on exploring mental health trends within a preschool demographic, despite the absence of specific threshold values for these dimensions. This methodological decision aligns with the objective to broadly investigate mental health trends, emphasizing the importance of considering variations within the normal range and the potential for future research to further refine the SDQ's application in varied settings.

#### 2.2.4 Demographic characteristics

The study incorporated various demographic attributes as covariates, including the children's age, gender, height, weight, body mass index (BMI), and family socioeconomic status (SES), aligning with earlier research protocols (6, 9, 11). The data concerning these variables, except for family SES, were sourced from kindergarten teachers. Family socioeconomic status was gauged through a questionnaire that parents completed, comprising inquiries regarding parental occupation, education, and monthly household income (41). Utilizing principal component data analysis, the SES index was calculated, and the socioeconomic status was subsequently categorized into high, medium, or low tiers based on the standard deviation.

#### 2.2.5 Statistical analysis

Participants were classified based on whether they met or did not meet the 24-h Movement Behaviour guidelines, as well as combinations thereof (e.g., meeting PA + screen time only, meeting PA + sleep duration only, meeting screen time + sleep duration only, and meeting all three guidelines). The sample data underwent descriptive statistical analyses, encompassing calculations of means, standard deviations (SD), and percentages.

For delving into the connection between preschool children's mental health and their adherence to the 24-h Movement Behaviour guidelines, Structural Equation Modeling analysis was conducted employing Mplus 8.0 (Muthén & Muthén, 2017) software. This analysis was adjusted for covariates including gender, age, BMI, and SES. The threshold for statistical significance in all analyses was set at *p* < 0.05.

## 3 Results

### 3.1 Descriptive analysis

Table 1 shows the descriptive statistics for preschool children's 24-h Movement Behaviour and mental health. Following the guidelines set forth by the World Health Organization (19), the preschoolers met the recommended total PA time but fell short of meeting the guidelines for MVPA. However, they did meet the guidelines for both sleep duration and screen time. Specifically, the average daily PA duration was 257 min, with MVPA accounting for 48.5 min. The children's daily sleep duration averaged 636.79 min, and their daily screen time amounted to 106.61 min.

**Table 1:** Descriptive statistics of the sample.

	Mean	Standard deviation
Age (year)	4.89	0.74
MVPA (min/day)	48.5	14.54
PA (min/day)	257.93	40.91
SP (min/day)	636.79	56.24
ST (min/day)	106.61	63.08
BMI	15.63	1.636
Prosocial behaviour	6.73	1.72
INTER	6.43	2.50
EXTER	6.043	2.09

PA, physical activity; MVPA, moderate-to-vigorous physical activity; SP, sleep duration; ST, screen time; BMI, body mass index; INTER, internalizing problems; EXTER, externalizing problems.

**Figure 2:**
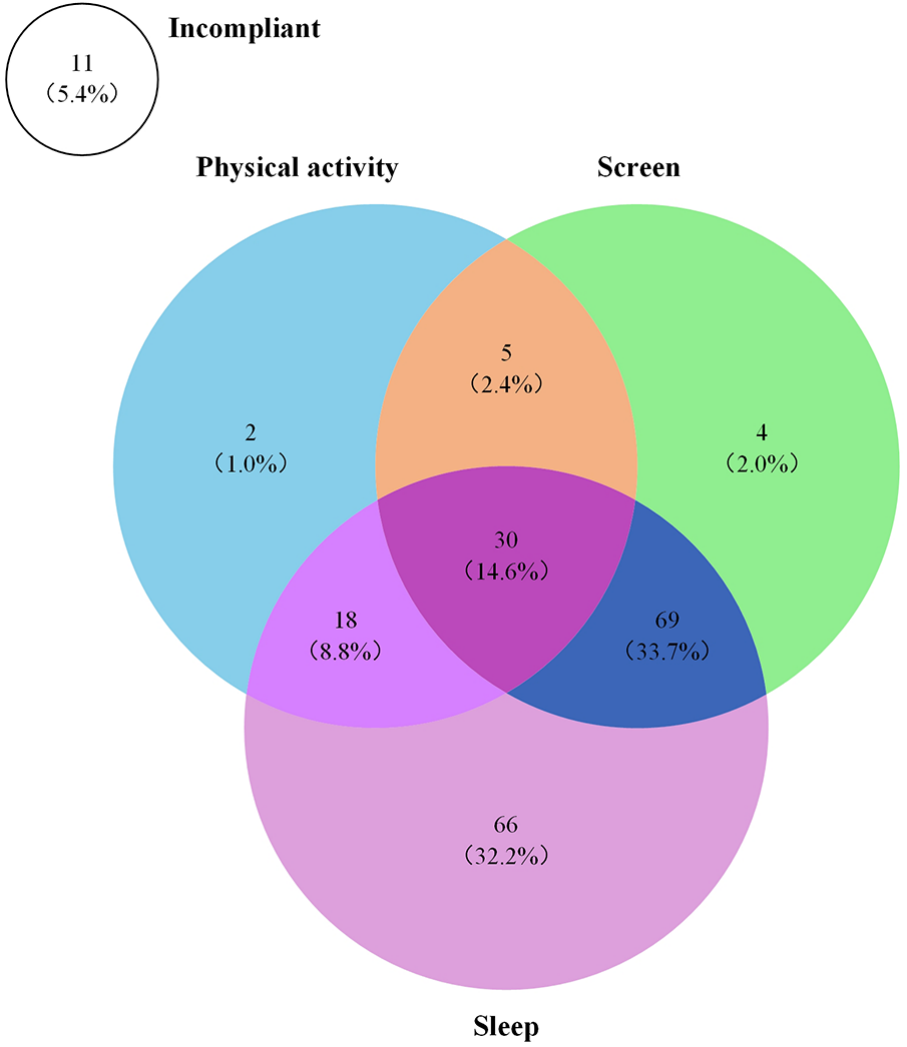
Proportion of participants meeting the 24-h Movement Behaviour guidelines in various combinations. The sum of each circle corresponds to the percentage of each recommendations met in the whole study sample (i.e., 26.8% physical activity, 89.3% sleep, 52.7% screen time, 5.4% non-compliance in the whole study sample).

Figure 2 displays a Venn diagram illustrating the proportion of preschoolers meeting the 24-h Movement Behaviour guidelines for various combinations of modalities. As depicted in the figure, the majority of preschoolers met the recommended guidelines for screen time (52.7%) and sleep duration (89.3%), while only 26.8% met the recommended guidelines for PA. Notably, 5.4% of preschoolers did not meet any of the recommended guidelines, and only 14.6% were able to meet all three recommended guidelines concurrently. Moreover, 2.4% met the combined PA and screen time recommendation, 8.8% met the combined PA and sleep duration recommendation, and 33.7% met the combined sleep duration and screen time recommendation.

### 3.2 Associations among meeting the guidelines and mental health

The initial model comprised 25 items, but the correlation index did not meet the standard (RMSEA = 0.07, CFI = 0.69, TLI = 0.66). To improve the model's fit, we eliminated items with low correlations (items 6, 11, 23, 7, 12, 22, 21, 25) and reconfigured the remaining 17 items into three dimensions: internalizing, externalizing, and prosocial behaviour. The adjusted second-round structure model demonstrated a significantly improved fit index (RMSEA = 0.04, CFI = 0.93, TLI = 0.91).

**Figure 3:**
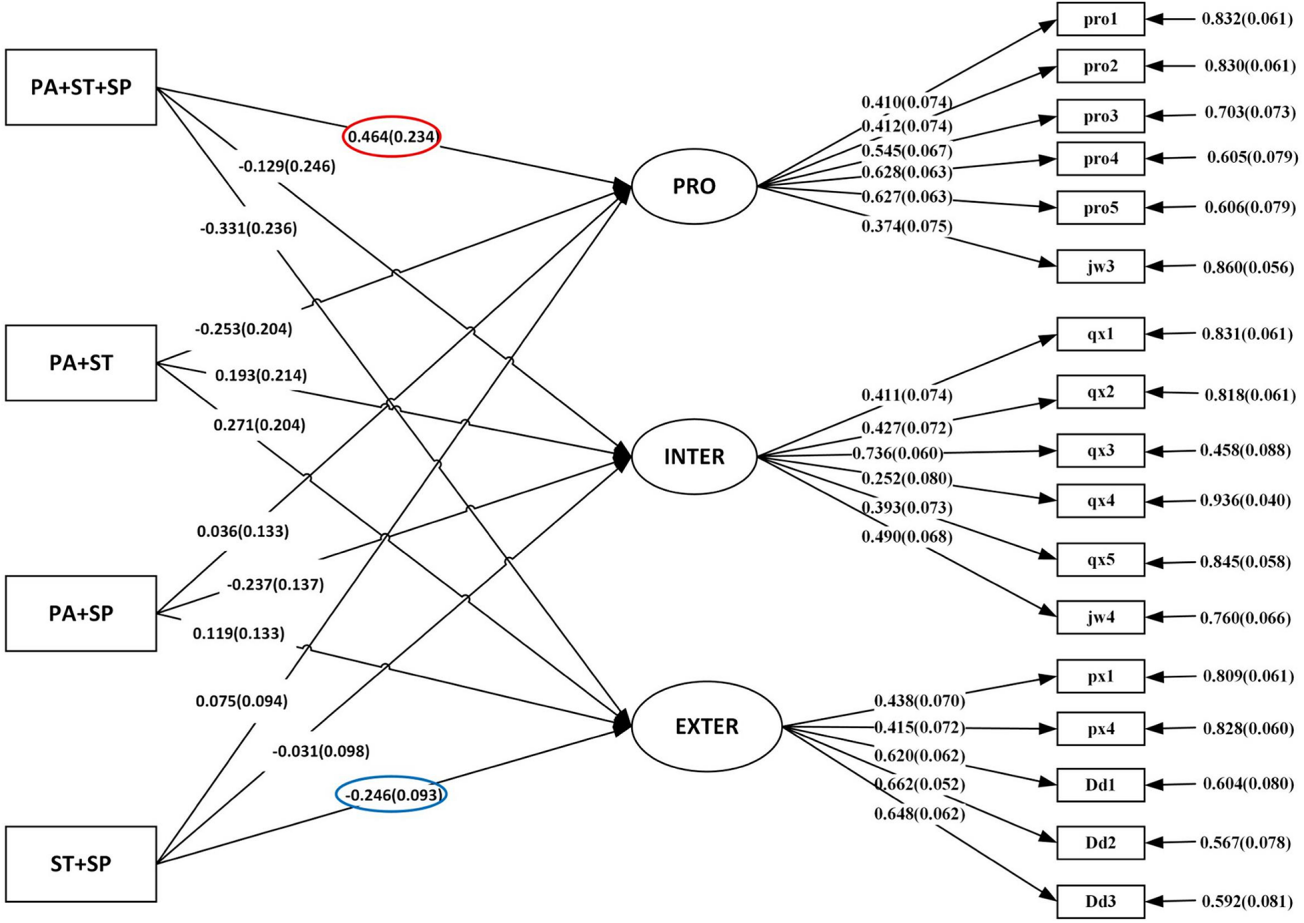
Structural modeling of the relationship between the combinations of met 24-h movement behaviour guidlines and mental health in preschoolers. PA, physical activity; SP, sleep; ST, sceen time; INTER, internalizing problems; EXTER, externalizing problems; PRO, prosocial behaviour. Colored with red line indicates significant positive relationship; Colored with blue line indicates significant negative relationship.

As seen from Table 2 and Figure 3, the relationship between adhering to individual guidelines for 24-h Movement Behaviour and mental health yielded compelling insights. Notably, children who met the PA guideline demonstrated a positive association with prosocial behaviour (*β* = 0.184; *p* < 0.05). Conversely, meeting the guidelines for screen time and sleep duration exhibited negative associations with externalizing problems (*β* = −0.207; *p* < 0.05 for screen time and *β* = −0.191; *p* < 0.05 for sleep duration).

**Table 2:** Relationship between meeting the 24-h Movement Behaviour guidelines and mental health.

	INTER		EXTER		PRO	
	*β*	*P*	*β*	*P*	*β*	*P*
PA	−0.124	0.144	0.069	0.401	**0** **.** **184**	**0**.**026**
ST	0.048	0.575	**−0**.**207**	**0**.**012**	0.089	0.286
SP	−0.129	0.129	**−0**.**191**	**0**.**018**	0.077	0.359
PA + ST + SP	−0.029	0.906	−0.331	0.16	**0**.**464**	**0**.**048**
PA + ST	0.193	0.365	0.271	0.185	−0.253	0.215
PA + SP	−0.237	0.084	0.119	0.371	0.036	0.788
ST + SP	−0.031	0.75	**−0**.**246**	**0**.**008**	0.075	0.427

PA, physical activity; SP, sleep duration; ST, screen time; INTER, internalizing problems; EXTER, externalizing problems, PRO, prosocial behaviour; Bold represents significant relationship.

In terms of the relationship between preschoolers adhering to the combined 24-h Movement Behaviour guidelines and their mental health, our data revealed significant outcomes. Preschoolers who met all three recommended guidelines displayed a positive association with prosocial behaviour (*β* = 0.464; *p* < 0.05). Moreover, meeting the guidelines for screen time and sleep duration demonstrated a negative association with externalizing problems (*β* = −0.246; *p* < 0.05). These findings provide valuable insights into the interplay between meeting multiple behavioural guidelines and its impact on the mental health of preschooler.

## 4 Discussion

### 4.1 Descriptive findings of compliance with the 24-h movement behaviour guidelines

The findings of this study unveil that a considerable majority of Chinese preschoolers adhere to the recommended guidelines for screen time at 52.7% and sleep duration at 89.3%, while only a modest portion meet the criteria for PA at 26.8%. Surprisingly, a mere 14.6% of preschoolers meet all three guidelines, with 5.4% failing to meet any. These outcomes are consistent with earlier research that also yielded similar results (7, 42, 43). Noteworthy is the substantial proportion of preschoolers in our study who conform to the prescribed sleep duration (89.3%), which correlates with figures from Canada (83.9%) (42) and Australia (88.7%) (43). However, our study showcases a lower percentage in terms of meeting PA guidelines compared to Canada (61.8%) (42) and Australia (93.1%) (43), signifying a deficiency in aligning with the 24-h Movement Behaviour guidelines.

It is noteworthy to highlight that certain European studies have reported high levels of adherence to PA guidelines. For instance, Collings conducted a survey involving 333 preschool children in England, revealing that 95.4% of them meet the recommended 180 min of PA per day (44). Discrepancies such as these could potentially be attributed to variations in accelerometer utilization and cut-off points. The Australian study, similar to ours in accelerometer usage, employed a combination of two cut-off points to evaluate PA behaviour in children (45, 46), including a threshold of 420 counts per 15 s (c/15 s) for MVPA. In contrast, our study adopted Butte et al.'s recommendation, using 212° c/15 s as the MVPA cut-off point, which might lead to a lower detection rate of MVPA and consequently a decline in the overall PA detection rate (33). It's crucial to note, however, that Butte's cut-off point has been validated for application within preschool populations (33). Another potential factor contributing to the relatively low adherence to PA guidelines in China could be the strong emphasis on academic pursuits over PA, as a result of the prevailing concept of “intellectual priority” held by many parents.

### 4.2 Relationships between preschooler's mental health and compliance with the 24-h movement behaviour guidelines

The principal aim of this study was to explore the connection between adhering to the 24-h Movement Behaviour guidelines and the mental well-being of preschool children, while concurrently identifying the combinations that yield more substantial benefits. Our findings have illuminated several significant insights. Specifically, we observed that individual screen time or sleep duration behaviours exhibited a negative correlation with externalizing problems in children. Moreover, the combination of adhering to both screen time and sleep duration guidelines was also linked to a reduction in externalizing problems. These findings suggest that adhering to the recommended sleep duration can potentially mitigate the occurrence of externalizing issues, encompassing hyperactivity and inattention. Furthermore, simultaneously adhering to both screen time and sleep duration guidelines seemed to confer a similar positive effect in diminishing externalizing problems. Although no statistically significant association emerged between PA and externalizing problems, a positive connection was discerned between PA and prosocial behaviour in children. Notably, meeting the 24-h Movement Behaviour guidelines in conjunction with adhering to the recommended screen time and sleep duration was positively correlated with prosocial behaviour among children. Given that prosocial behaviour is a positive facet (47), these findings underscore the benefits of adhering to PA recommendations for children's mental health. Moreover, the concurrent compliance with PA, screen time, and sleep duration recommendations was associated with improved mental health outcomes.

Primarily, our study has yielded evidence that insufficient sleep duration is associated with a heightened occurrence of externalizing behaviours, which include infractions and aggressive behaviours. This finding aligns seamlessly with outcomes reported in earlier research studies (48–50). The significance of adequate and high-quality sleep, recognized for its close correlation with both physical and mental health, cannot be underestimated (9). The impact of inadequate sleep extends to the brain's capacity to regulate and express emotions, thereby impairing the development of executive functions and giving rise to heightened irritability, anger, and impulsiveness (51, 52). Furthermore, inadequate sleep brings about daytime sleepiness and fatigue, curtailing the time available for PA and ultimately casting an adverse impact on both physical and mental well-being (53). Moreover, the proliferation of portable electronic devices has played a pivotal role in the upsurge of screen time among children. The ease of access to these devices has enabled children to bring them into their bedrooms, leading to delayed sleep initiation and reduced sleep duration. Consequently, this dynamic exerts an influence on both physical and mental health (54). To underscore this, a study encompassing 9 to 10-year-old children unveiled that increased screen time was linked to a heightened prevalence of externalizing behaviours (49).

Moreover, our study unveiled a significant and inverse correlation between meeting the combined screen time and sleep duration recommendation (screen time + sleep duration) and externalizing problems among preschoolers. This finding underscores that adhering to the screen time + sleep duration recommendation proves to be notably effective in mitigating externalizing problems. Notably, this outcome resonates with prior research findings (26, 50, 55), even though these previous studies focused on participants aged 9–12 years (50, 55). It is worth considering that emotional and behavioural challenges can manifest differently across various age groups (56), thereby accentuating the importance of delving into the mental health of children spanning different age brackets. To our knowledge, only a single study conducted in Canada has examined the relationship between meeting screen time and sleep duration recommendations and externalizing problems in younger children (3 years old) (26). Adding to the complexity are policy and cultural distinctions between Western nations and China. A cross-sectional investigation delving into the adherence to 24-h Movement Behaviour guidelines and their impact on the mental health of Chinese preschoolers can potentially establish a more comprehensive theoretical foundation within the realm of health promotion. In addition, our study brought to light a noteworthy positive connection between PA and preschool children's prosocial behaviour. This observation underscores that adhering to PA recommendations leads to an elevation in prosocial behaviour. Prosocial behaviour is an essential facet of social interaction, contributing to the establishment and sustenance of positive interpersonal relationships while fostering social unity (57). Prior research has validated that active engagement in PA tends to foster improved prosocial behaviour and concurrently diminish social interaction difficulties (47, 58). PA not only affords opportunities for interaction with peers but also cultivates essential social skills and bolsters problem-solving aptitudes, culminating in the advancement of prosocial behaviour (59). This finding consequently supports the reasoning for Physical Education educators to place emphasis on teamwork and problem-solving competencies within their instructional framework.

Furthermore, our study elucidated that meeting the joint recommendations for PA, screen time, and sleep duration concurrently correlated with enhanced prosocial behaviour in comparison to not meeting any of these recommendations. Numerous research endeavors have consistently highlighted that fulfilling all three guidelines not only leads to improvements in anxiety, depression, and life satisfaction but also augments prosocial behaviour (20, 22, 24). Children adhering to two or more of these recommendations have also demonstrated heightened social functionality across domains like externalizing problems, internalizing problems, and prosocial behaviour, coupled with lower stress levels (48). Hence, the logical inference emerges that the children in our study who fulfilled the combined PA + screen time + sleep duration recommendation exhibited superior prosocial behaviour. Nevertheless, it's noteworthy that merely 14.6% of children in our study concurrently fulfilled all three behavioural recommendations, while the majority met only one or two of them. It is plausible that this limited sample size could potentially impact the analysis of the relationship between fulfilling all three behavioural recommendations and mental health outcomes. Nonetheless, two longitudinal investigations have underscored that compliance with comprehensive guidelines doesn't necessarily correspond to improved psychosocial well-being over the course of one year (60) to three years (61). In light of this, it is prudent for future research endeavors to extend their scope beyond examining horizontal associations, and instead, allocate due emphasis on longitudinal interventions as well.

### 4.3 Study strengths and limitations

This study serves as a pioneering exploration of the connection between 24-h Movement Behaviour and mental health in Chinese preschool children, with a distinctive emphasis on delineating the most advantageous combinations of behaviours that significantly contribute to children's mental well-being. The research boasts notable strengths, including the objective assessment of children's PA through accelerometers and the implementation of advanced statistical analysis techniques to interpret the amassed data. Nonetheless, it is imperative to acknowledge several limitations inherent to this study that merit thoughtful deliberation. To commence, it's essential to acknowledge that the evaluation of children's sleep duration, screen time, and mental health was reliant on questionnaires completed by parents, potentially introducing measurement biases. Acknowledging the potential discomfort associated with wearing an accelerometer on the right hip during sleep (62), a questionnaire was employed for sleep duration assessment, although it failed to encompass the measurement of sleep efficiency. Subsequent investigations should contemplate the incorporation of objective tools for appraising sleep in preschool children, thereby augmenting measurement precision. Moreover, this study's emphasis on sedentary behaviour primarily via screen time could inadvertently overshadow the impact of non-screen sedentary behaviour in children's everyday routines (11). To gain a more comprehensive insight into its potential sway on mental health outcomes, a broader analysis of sedentary behaviour patterns becomes imperative. Furthermore, the cross-sectional framework adopted in this study precludes the determination of causal relationships between movement behaviours and mental health outcomes. To establish causality, forthcoming research could embrace longitudinal designs, facilitating the tracking of changes over time. Lastly, the study's confined geographical range, conducted exclusively in a substantial city in south-central China, might restrict the generalizability of its findings to other locales or population groups. To establish a more extensive relevance, future investigations should contemplate the inclusion of diverse samples from various regions. Despite these acknowledged limitations, the current study constitutes a notable advancement in unraveling the intricate interplay between 24-h Movement Behaviour and mental health within the context of Chinese preschool children. The incorporation of accelerometers and meticulous statistical analyses not only bolsters the credibility of the findings but also accentuates the significance of the research. Undertaking proactive measures to address the outlined limitations in subsequent research endeavors holds the potential to amplify the field's comprehension of the intricate nexus between movement behaviours and mental well-being in this particular age cohort.

## 5 Conclusion

This study emphasizes the critical gap in adherence to the 24-h Movement Behaviour guidelines among Chinese preschoolers, with only 14.6% meeting the comprehensive guidelines for PA, sleep duration, and screen time. The findings highlight the positive impact of PA on fostering prosocial behaviour and the significant role that adherence to sleep duration and screen time guidelines plays in mitigating externalizing problems among this age group. These results underscore the importance of a holistic approach to Movement Behaviour guidelines to improve mental health outcomes in preschool children.

## Data Availability

The raw data supporting the conclusions of this article will be made available by the authors, without undue reservation.
